# Moral injury and suicide ideation among combat veterans: The moderating role of self-disclosure

**DOI:** 10.1192/j.eurpsy.2023.2379

**Published:** 2023-07-19

**Authors:** Y. Levi-Belz

**Affiliations:** 1 Ruppin Academic Cener, Emek hefer, Israel

## Abstract

**Introduction:**

Modern warfare in a civilian setting may expose combatants to severe moral challenges. Whereas most of these challenges are handled effectively, some *potentially morally injurious events* (PMIEs) may have deleterious psychological effects on the combatants, such as suicide ideation (SI). Self-disclosure, which includes sharing distressing thoughts and emotions, has been recognized as a protective factor against SI in the aftermath of stressful events.

**Objectives:**

The current study is the first to examine the moderating role of self-disclosure in the relationship between PMIEs exposure and SI among combat veterans.

**Methods:**

A sample of 190 recently discharged Israeli combat veterans completed validated self-report questionnaires in a cross-sectional design study, tapping combat exposure, PMIEs, depressive symptoms, SI, and self-disclosure.

**Results:**

PMIE dimensions, and self-disclosure significantly contributed to current SI. Importantly, the moderating model indicated that self-disclosure moderated the link between PMIE-Self and current SI , as PMIE-Self and current SI were more strongly associated among veterans with low levels of self-disclosure than among high self-disclosing veterans.

**Conclusions:**

Self-disclosure, as a factor promoting a sense of belongingness, interpersonal bonding, and support, might reduce SI risk following PMIE exposure. Various mechanisms accounting for these associations are suggested, and clinical implications of these interactions are discussed.

**Disclosure of Interest:**

Y. Levi-Belz Shareolder of: no, Grant / Research support from: no, Consultant of: no, Employee of: no, Paid Instructor of: no, Speakers bureau of: no

